# CuO/ZnO Heterojunction Nanorod Arrays Prepared by Photochemical Method with Improved UV Detecting Performance

**DOI:** 10.3390/nano9050790

**Published:** 2019-05-23

**Authors:** Jieni Li, Tingting Zhao, Mandar M. Shirolkar, Ming Li, Haiqian Wang, Henan Li

**Affiliations:** 1 International Collaborative Laboratory of 2D Materials for Optoelectronics Science and Technology of Ministry of Education, College of Physics and Optoelectronic Engineering, Shenzhen University, Shenzhen 518060, China; jnli91@szu.edu.cn; 2 Hefei National Laboratory for Physical Sciences at the Microscale, University of Science and Technology of China, Hefei 230026, Anhui, China; zhaott27@mail.ustc.edu.cn (T.Z.); mmshirolkar@gmail.com (M.M.S.); seagullc@ustc.edu.cn (M.L.); 3 Symbiosis Center for Nanoscience Center and Nanotechnology, Symbiosis International, Deemed University, Lavale, Pune 412115, India; 4 College of Electronic Science and Technology, Shenzhen University, Shenzhen 518060, China

**Keywords:** CuO/ZnO heterojunction NRs, photochemical deposition, improved UV detecting performance, Cu_2_(NO_3_)(OH)_3_/ZnO precursor

## Abstract

CuO/ZnO heterojunction nanorod arrays were synthesized using a facile photochemical deposition strategy. The morphology of CuO was related to the concentration of Cu^2+^ in the Cu(NO_3_)_2_ solution, UV illumination time, and the air annealing temperature. A possible reaction mechanism was proposed. In the photochemical deposition process, the OH^−^ was generated in the vicinity of the ZnO nanorod arrays and reacted with Cu^2+^ and NO_3_^−^ in the solution to form Cu_2_(NO_3_)(OH)_3_/ZnO heterojunction nanorod arrays firstly, which were converted into CuO/ZnO heterojunction nanorod arrays completely after air annealing at a low temperature. The fabricated CuO/ZnO heterojunction nanorod arrays exhibits a well-defined rectifying characteristic and an improved photo-response performance compared with pure ZnO nanorod arrays.

## 1. Introduction

ZnO is an attractive oxide semiconductor because of its tunable bandgap, stability at high temperature, and ability to be operated in hostile environments, which makes it one of the most important materials for UV photodetectors [1,2,3,4,5]. Moreover, ZnO nanorod arrays (NRs) are especially attractive due to their excellent properties, such as large ratio of surface area to volume and outstanding charge carrier transport for ensuring high efficiency and sensitivity [6]. However, the UV photodetectors fabricated from pure ZnO NRs often have low photosensitivity due to the photo-generated carrier fast recombination, and a long response time due to the absorption of oxygen molecules [7,8,9].

To solve these problems and make the UV detecting properties of ZnO NRs further improved, many kinds of materials have been explored to modify the surface of ZnO NRs, such as polymers [10], noble metal nanoparticles [11], and oxide semiconductors [12,13,14]. Wang et al. [15] used Ag nanoparticles decorating ZnO NR surfaces to facilitate the separation of photogenerated carriers and improve the photo response performance of the UV photodetectors. Chen et al. [16] modified a 3-aminopropyltrimethoxysilane monolayer on the surface of the ZnO film to reduce the negative influences of adsorbed oxygen molecules and improve the performance of the ZnO UV sensor. Among various oxide semiconductor materials, CuO is a common metal oxide, which is an intrinsic p-type semiconductor and shows a small band gap (1.2 eV) [17]. So far, CuO has been substantially explored for various fields of applications, such as field emitters, photocatalysis, and gas sensing [18,19,20,21,22,23]. Moreover, CuO has the advantage of its large abundance in nature, its low cost, its inability to pollute, and its simple synthesis like ZnO [24]. Previously, CuO/ZnO heterojunction nanostructures have been extensively studied in photocatalytic reactions [25,26,27]. The CuO of CuO/ZnO heterojunctions were often prepared by radio-frequently (RF) magnetron sputtering techniques or electrochemical deposition (ECD) [28,29,30,31]. These methods often need high temperature, high cost, and a long time. Thus, it is of great significance to develop a facile method to prepare a CuO/ZnO heterojunction, which has a low cost, simple process, is environmentally friendly and has a high product yield. The photochemical deposition method is easy, low cost, and its process is controllable. In a photochemical deposition process, the redox reactions of aqueous chemical species conduct on the surface of photocatalytic solids under light illumination and the target products are fabricated [14,15]. Taking these merits into account, the photochemical deposition method is considered to be an outstanding synthesis strategy for noble metal nanoparticles or semiconductor oxides on ZnO NRs. In addition, photodetectors based on CuO/ZnO heterostructures often show low photo-response performance, including low photosensitivity and a long response time. Morphology is an important factor that affects the photo-response performance of a CuO/ZnO based photodetector. Furthermore, the morphology of a CuO /ZnO heterostructure is often dominated by the precursor. In many previous works, CuO in a CuO/ZnO heterostructure fabricated from Cu_2_(NO_3_)(OH)_3_ precursor has not been reported [28,29,30,31]. Therefore, taking the precursor into account, further explorations for improving the photo-response performance of the device is very important.

In this study, we report CuO/ZnO hierarchical nanostructures heterojunction NRs that consist of 1D ZnO nanorods (NRs) and flower-like CuO nanostructures, which were successfully synthesized by a facile photochemical deposition method and a following air annealing process. The impact of Cu^2+^ ion concentration, UV illumination time, and annealing temperature on the fabricated CuO/ZnO NRs were discussed in detail. All the devices based on CuO/ZnO heterojunction NRs display well-defined rectifying characteristics. Their UV photo-response performance was also measured. All the devices exhibit improved photosensitivity and a shorter reset time compared with the device based on pure ZnO NRs. Among them, CuO/ZnO NRs fabricated from a 50 mM Cu^2+^ ion concentration, 20 min UV illumination time, and 300 °C annealing time shows optimum UV detecting performance.

## 2. Experiment

**Preparation of the CuO/ZnO heterojunction NRs:** The preparation of CuO/ZnO heterojunction NRs included three steps: (1) synthesis of ZnO NRs, (2) preparation of Cu_2_(NO_3_)(OH)_3_/ZnO heterojunction NRs precursor, and (3) preparation of CuO/ZnO heterojunction NRs through the air-annealing process of a Cu_2_(NO_3_)(OH)_3_/ZnO heterojunction NR precursor.

**Synthesis of ZnO NRs:** The ZnO NRs were synthesized by a simple hydrothermal method [32]. A magnetron sputtering system was used to deposit an AZO (Al doped ZnO) seed layer (400 nm) on a glass substrate [33]. A Hall measurement shows that the AZO film has a sheet resistance of 3.4 × 10^−3^ Ω cm, suggesting the feasibility of AZO as a seed layer and transparent electrode. The AZO coated substrate was immersed into an equal mole of zinc acetate (Zn(Ac)_2_·2H_2_O) and hexamethylenetetramine (C_6_H_12_N_4_, HMT) solution with a concentration of 0.02 M in a Teflon liner stainless-steel autoclave. Then, the autoclave was kept in a 90 °C oven for 12 h to grow ZnO NRs. After the growth process was finished, the resulting ZnO NRs were taken out and cleaned with deionized water and ethanol several times. The dried ZnO NRs showed a white appearance.

**Preparation of Cu_2_(NO_3_)(OH)_3_/ZnO heterojunction NR precursors:** The Cu_2_(NO_3_)(OH)_3_/ZnO precursor was deposited by a simple photochemical process. Copper nitrate (Cu(NO_3_)_2_·3H_2_O) was added into deionized water to form a Cu(NO_3_)_2_ solution with a concentration ranging from 2 to 100 mM. Then, the ZnO NRs were immersed into the above solution and irradiated by UV light (365 nm, 2.0 mW/cm^2^). The reaction was conducted at room temperature and the reaction time ranged from minutes to 1 h. After the reaction finished, the samples were washed several times and dried in a 60 °C oven. The samples showed a blue appearance.

**Preparation of CuO/ZnO heterojunction NRs:** The CuO/ZnO heterojunction NRs were prepared by annealing the as-prepared blue Cu_2_(NO_3_)(OH)_3_/ZnO precursors at 200, 300, and 400 °C in air for 2 h. The oven was heated with a ramping rate of 2 °C/min. The color of the final CuO/ZnO heterostructure NRs was black. Ag paste was used to prepare electrode.

**Characterization:** X-ray diffraction (XRD, a Panalytical X’PERT PRO diffractometer using Cu-Kα radiation, Almero, The Netherlands) was used to characterize the crystalline of the samples. The morphology was measured by scanning electron microscopy (SEM, JSM-6700F, Tokyo, Japan). Transmission electron microscopy (TEM) and high resolution transmission electron microscopy (HRTEM) were measured by a JEOL JEM-ARF200F atomic resolution analytical microscope (JEOL, Tokyo, Japan) with an accelerating voltage of 200 kV. Thermogravimetry (TG), and Differential Scanning Calorimetry (DSC) (STA449F3, NETZSCH, Bavaria, Germany) were used to study the decomposition process of Cu_2_(NO_3_)(OH)_3_. The photo-response performance was measured under 365 nm UV light illumination. The light intensity is 2.0 mW/cm^2^, calibrated by a PM100A light power meter (Thorlabs, Shanghai, China) with a UV-enhanced Si detector. The UV light was from the CuO side. Keithley 2420C Source Meter (Keithley, Shanghai, China) was used to record the I-V characteristics curves. Keithley 2000 multimeter (Keithley, Shanghai, China) was used to measure the voltage of a 1 MΩ resistance in series with the photodetector. Because the resistance of the photodetector was large enough, so the current across the 1 MΩ resistance can be used to represent the photocurrent.

## 3. Results and Discussion

### 3.1. Characteristics of Materials

**Figure 1:**
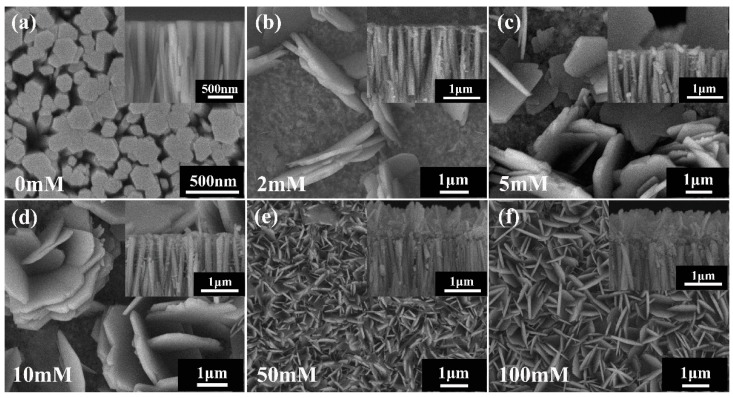
Surface and cross section morphologies of the ZnO nanorod arrays (NRs) and the Cu_2_(NO_3_)(OH)_3_/ZnO NR precursor. 365 nm UV illumination time was fixed at 20 min and the light intensity is 2 mW/cm^2^. (**a**) pure ZnO NRs. (**b**–**f**) Cu_2_(NO_3_)(OH)_3_/ZnO NR precursors deposited by the photochemical method in solutions with Cu^2+^ ion concentration of 2 mM, 5 mM, 10 mM, 50 mM, and 100 mM, respectively.

In order to determine the effect of Cu^2+^ ion concentration on the photochemical synthesis of the Cu_2_(NO_3_)(OH)_3_/ZnO heterojunction NRs, we examined the morphology of the products prepared from solutions with various Cu^2+^ concentrations of 2, 5, 10, 50, and 100 mM. Figure 1a exhibits the surface and cross section morphologies of pure ZnO NRs synthesized through a hydrothermal reaction. It is obvious that ZnO NRs grow perpendicularly on the AZO coated substrate and have a smooth surface. The ZnO NRs are about 2.0 μm long and the diameter varies from 100 nm to 300 nm. Moreover, the ZnO NRs show a hexagonal cross section, which indicates the hexagonal wurtzite structure and preferential orientation in the c-axis direction. Figure 1b–f shows the surface and cross-section morphologies of the Cu_2_(NO_3_)(OH)_3_/ZnO heterojunction NRs prepared in various Cu^2+^ ion concentration Cu(NO_3_)_2_ aqueous solutions with 20 min UV illumination. When the Cu^2+^ ion concentration is 2 mM, the surface of ZnO NRs are mostly covered by a thin layer of Cu_2_(NO_3_)(OH)_3_ nanoparticles, which are suitable to act as a Cu_2_(NO_3_)(OH)_3_ seed for further growth. Moreover, some immature flower-like Cu_2_(NO_3_)(OH)_3_ nanostructures formed on the surface of ZnO NRs. These nanostructures are composed of nanosheet-shape building blocks, which have a smooth surface. By further increasing Cu^2+^ ion concentration, the sheet-like Cu_2_(NO_3_)(OH)_3_ nanostructures deposited on the surface of ZnO NRs become more dense, which can be clearly seen in the SEM images. It is noteworthy that the Cu_2_(NO_3_)(OH)_3_ eventually covers the whole surface of ZnO NRs as the Cu^2+^ ion concentration rises to 50 mM. When the Cu^2+^ ion concentration increases to 100 mM, the Cu_2_(NO_3_)(OH)_3_ layers on the ZnO NRs exhibit a larger size than that of the Cu_2_(NO_3_)(OH)_3_ deposited from the solution with 50 mM Cu^2+^ ion concentration.

**Figure 2:**
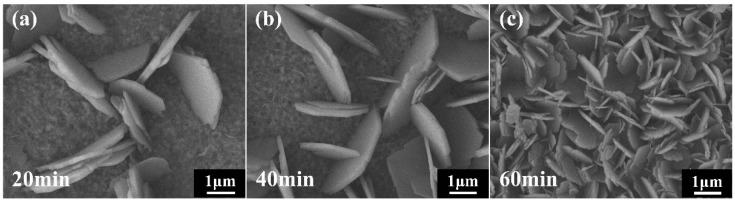
The surface morphology of the Cu_2_(NO_3_)(OH)_3_/ZnO precursors prepared at a fixed Cu^2+^ ion concentration (2 mM) and various illumination time: (**a**) 20 min, (**b**) 40 min and (**c**) 60 min.

Further, the effects of UV illumination time on the surface morphology of the Cu_2_(NO_3_)(OH)_3_/ZnO precursor were investigated. Because the surface of the samples prepared from 50 mM Cu^2+^ concentration solution is fully covered by Cu_2_(NO_3_)(OH)_3_ in a short time and the effect of UV illumination time cannot be clearly observed. Thus, we chose the sample prepared from a low Cu^2+^ concentration solution to study the effect of UV illumination time. The reactions are conducted at a fixed Cu^2+^ ion concentration of 2 mM and UV illumination times of 20 min and 40 min to 60 min. With increasing UV illumination time, the sheet-like Cu_2_(NO_3_)(OH)_3_ nanostructures deposited on the ZnO NRs became more dense. The change can be clearly observed in the surface morphology images, as shown in Figure 2a–c. It is noteworthy that the ZnO NR surface can be fully covered with the Cu_2_(NO_3_)(OH)_3_ layer as the UV illumination time rises to 60 min.

**Figure 3:**
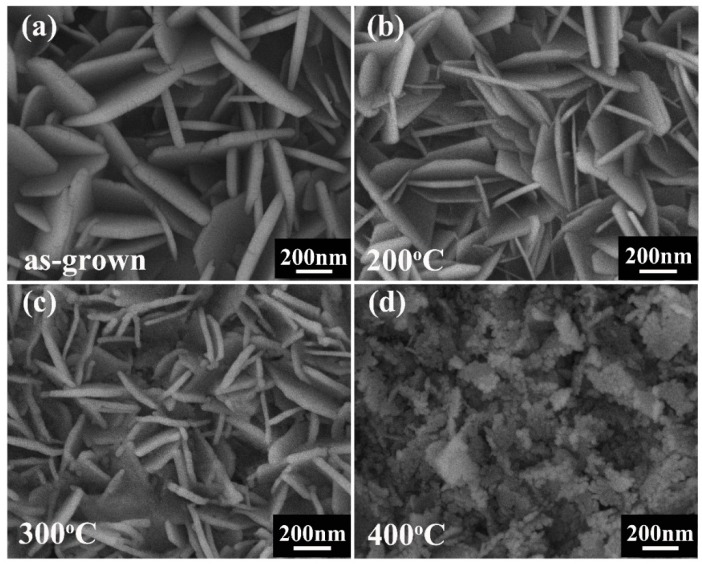
(**a**) The SEM image of the as-grown Cu_2_(NO_3_)(OH)_3_/ZnO NRs fabricated with 50 mM Cu2+ ion concentration and 20 min UV illumination time. (**b**) The SEM images of the Cu_2_(NO_3_)(OH)_3_/ZnO NRs annealed at 200 °C. (**c**,**d**) The SEM images of CuO/ZnO annealed at 300 °C and 400 °C, respectively.

We also studied the dependence of the morphology of the Cu_2_(NO_3_)(OH)_3_/ZnO NR nanostructures (50 mM Cu^2+^ ion concentration and 20 min UV illumination time) on annealing temperature. The Cu_2_(NO_3_)(OH)_3_/ZnO NR precursor prepared with 50 mM Cu^2+^ concentration and 20 min UV illumination time was annealed at 200, 300, and 400 °C for 1 h in the air. There is no obvious change from the ZnO NRs after thermal annealing at 400 °C in the air atmosphere (Figure S1, ESI), which is consistent with Dai’s report [14]. The surface morphologies of the as grown Cu_2_(NO_3_)(OH)_3_/ZnO heterojunction NR precursor and the samples annealing at different temperatures are shown in Figure 3. The surface morphology of the sample annealing at 200 °C in Figure 3b is similar to the as-grown Cu_2_(NO_3_)(OH)_3_/ZnO precursor (Figure 3a), except that the individual nanosheet of the building blocks becomes thinner. In addition, the sample annealing at 200 °C shows a gray appearance, which indicates that a few of the Cu_2_(NO_3_)(OH)_3_ nanostructures begin to convert to CuO. With the annealing temperature rising to 300 and 400 °C, the two samples show a black appearance and Cu_2_(NO_3_)(OH)_3_ is converted to CuO. The SEM morphology of the sample annealing at 300 °C, as shown in Figure 3c, shows obvious change because of the decomposition of Cu_2_(NO_3_)(OH)_3_. The nanosheet–shape structure is not smooth and many nanoparticles appear. For the sample annealing at 400 °C, these nanoparticles become denser and the nanosheet-shape nanostructures disappear, which is shown in Figure 3d. Therefore, it can be concluded that the annealing temperature has quite an impact on the morphology of the samples. Among all the samples, the sample annealed at 300 °C not only keeps the nanosheet morphology of the Cu_2_(NO_3_)(OH)_3_ precursor but also shows a good crystalline quality.

All these results show that the growth process of the CuO/ZnO heterojunction is dependent on the Cu^2+^ ion concentration of the Cu(NO_3_)_2_ solution, UV illumination time, and the annealing temperature.

**Figure 4:**
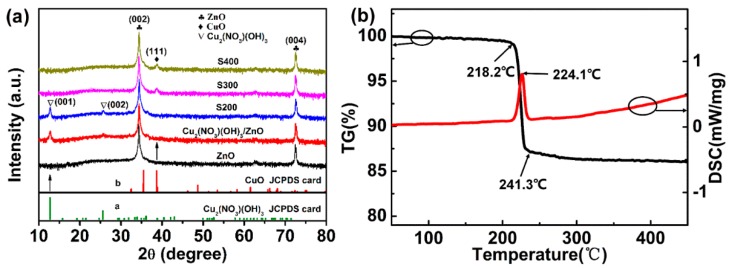
(**a**) XRD of the ZnO NRs, Cu_2_(NO_3_)(OH)_3_/ZnO NR precursor and Cu_2_(NO_3_)(OH)_3_/ZnO NRs annealed at 200, 300, and 400 °C. The reference patterns of Cu_2_(NO_3_)(OH)_3_ (JCPDS card no. 45-0594) and CuO (JCPDS card no. 48-4548). (**b**) The TG-DSC analysis of the Cu_2_(NO_3_)(OH)_3_/ZnO NR precursor fabricated with 50 mM Cu^2+^ ion concentration and 20 min UV illumination time.

The XRD pattern was used to analyze the phase structure of the samples before and after annealing (Figure 4a). All the samples show (002) and (004) diffraction peaks of wurtzite ZnO, further confirming the c-axis preferential orientation. The Cu_2_(NO_3_)(OH)_3_/ ZnO NR precursor prepared at room temperature displays two small peaks, which are (001) and (002) crystal plane of Cu_2_(NO_3_)(OH)_3_ (JCPDS card no. 45-0594). The weak intensity results from the small size of the as-synthesized Cu_2_(NO_3_)(OH)_3_ nanostructures in the specific crystal orientation. After annealing in air at 200 °C, the Cu_2_(NO_3_)(OH)_3_ phase was still maintained, and no peaks of CuO were observed. It can be concluded that only a few of the Cu_2_(NO_3_)(OH)_3_ nanostructures are converted to CuO, which cannot be detected by XRD due to its low content. Thus, the appearance changes from blue to gray while the sample stills shows an XRD diffraction peak of Cu_2_(NO_3_)(OH)_3_. With the annealing temperature rising to 300 and 400 °C, the peaks of Cu_2_(NO_3_)(OH)_3_ disappear, and a small peak of CuO (111) crystal plane (JCPDS card no. 48-1548) appears, which reveals that Cu_2_(NO_3_)(OH)_3_ completely converts to CuO. No other impurity diffractions were found.

The TG and DSC curves are measured to study the decomposition of the Cu_2_(NO_3_)(OH)_3_/ZnO NR precursor fabricated with 50 mM Cu^2+^ concentration and a 20 min UV illumination time, as shown in Figure 4b. The decomposition temperature ranges from 218.2 °C to 241.3 °C. The DSC curves shows a single endothermic peak centered at 224.1°C, indicating the one-step decomposition process of Cu_2_(NO_3_)(OH)_3_. Theoretically, the weight loss for the conversion of Cu_2_(NO_3_)(OH)_3_ to CuO is 33.17%. Here the weight loss is only 13.17%, which is due to the presence of ZnO NRs. In the whole process, the weight of ZnO has no change. Combined with the XRD analysis, the resultant is monoclinic CuO crystal. The thermal analysis results of the decomposition reaction are in accordance with many reports [34,35].

**Figure 5:**
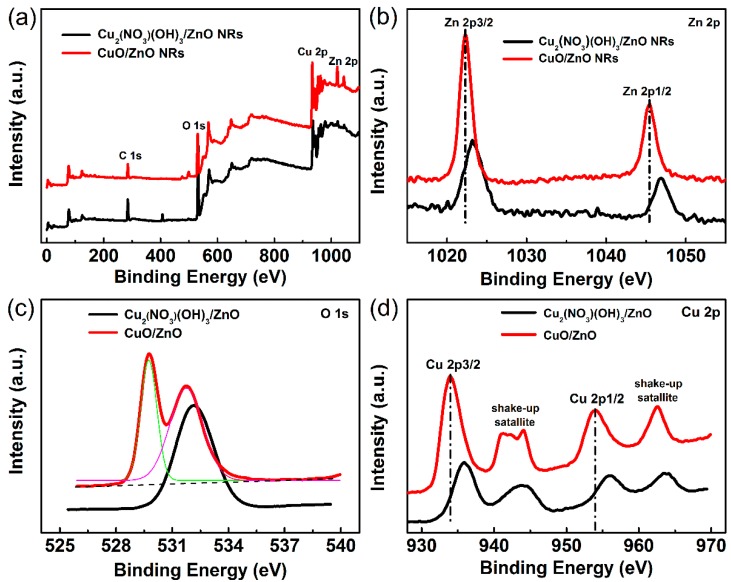
(**a**) The full survey XPS spectra, high resolution XPS spectra of (**b**) Zn 2p, (**c**) O 1s and (**d**) Cu 2p of the Cu_2_(NO_3_)(OH)_3_/ZnO and 300 °C annealing CuO/ZnO heterojunction NRs.

Furthermore, the phase transformation and chemical states of the as-synthesized Cu_2_(NO_3_)(OH)_3_/ZnO (50 mM Cu^2+^ ion concentration and 20 min UV illumination time) and CuO/ZnO heterojunction NRs were studied by XPS. The full survey of the two samples confirms the existence of Zn, Cu, O, and C elements (Figure 5a). Figure 5b–d shows the high resolution XPS spectra of Zn 2p, Cu 2p, and O 1s of the two samples. The XPS spectra of Zn 2p shows intense doublet peaks centered at 1022.3 and 1045.5 eV in the CuO/ZnO heterojunction NRs and 1023.4 eV and 1046.8 eV in the Cu_2_(NO_3_)(OH)_3_/ZnO heterojunction NRs, indicating the +2 oxidation state of Zn. Moreover, the Zn 2p_1/2_ and Zn 2p_3/2_ peaks show a splitting of ~23 eV, consistent with the value in existing literature [36]. In contrast with the Cu_2_(NO_3_)(OH)_3_/ZnO heterojunction, the peaks of Zn 2p in the CuO/ZnO heterojunction shift towards low binding energies, which result from the weak interaction of CuO and ZnO. Furthermore, the O 1s core level spectrum can be resolved into two O 1s peaks in CuO/ZnO heterojunction NRs. The 529.7 eV peak corresponds to the lattice binding of O^2−^ with Cu, while the peak at 531.8 eV is due to oxygen adsorbed on the CuO surface. For the O 1s spectrum of the Cu_2_(NO_3_)(OH)_3_/ZnO heterojunction NRs, there is only one broad peak centered at 532 eV, which should be attributed to surface adsorbed oxygen and oxygen species in the Cu_2_(NO_3_)(OH)_3_ precursor. The Cu 2p spectrum of the CuO/ZnO sample shows two peaks centered at 954 eV (Cu 2p_1/2_) and 934 eV (Cu 2p_3/2_). The sample also shows two satellites (asterisks) on the higher binding energy side of Cu 2p_1/2_ and Cu 2p_3/2_, which is consistent with the reported literature [26,37]. Compared with the Cu_2_(NO_3_)(OH)_3_/ZnO sample, the peaks in the Cu 2p spectrum of the CuO/ZnO sample shift towards lower binding energy, indicating the change of the chemical state of the Cu ions.

**Figure 6:**
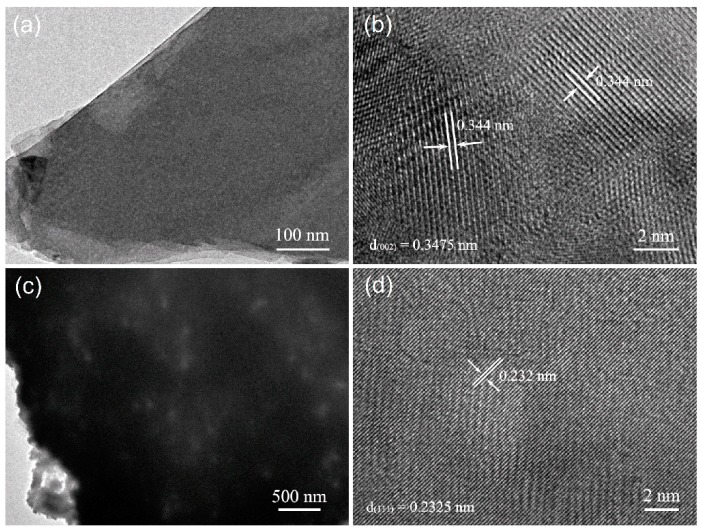
(**a**) The TEM and (**b**) HRTEM images of the as-grown Cu_2_(NO_3_)(OH)_3_ fabricated with 50 mM Cu^2+^ ion concentration and 20 min UV illumination time. (**c**) The TEM and (**d**) HRTEM images of the CuO annealed at 300 °C.

We further measured the TEM and HRTEM of the Cu_2_(NO_3_)(OH)_3_ (50 mM Cu^2+^ and 20 min UV illumination time) and CuO (300 °C annealing) samples, as shown in Figure 6. The samples were scraped from the substrates and ultrasonic dispersed in ethanol. In this process, Cu_2_(NO_3_)(OH)_3_ was separated from the ZnO nanorod. Figure 6a shows the TEM image of the as-prepared Cu_2_(NO_3_)(OH)_3_ nanosheet, which is consistent with the result of SEM. The crystalline structure of Cu_2_(NO_3_)(OH)_3_ is shown in its HRTEM image (Figure 6b). The atomic spacing is 0.344 nm, in agreement with the (002) plane of Cu_2_(NO_3_)(OH)_3_. In Figure 6c, CuO still keeps the nanoplate structure after 300 °C annealing. It can also be found that there are many pores in the nanoplate. The HRTEM image of CuO is shown in Figure 6d. The sample shows a good crystalline quality and the plane distance of CuO is 0.232 nm, which is in accordance with the (111) plane of CuO.

Combined with above experimental results, we propose a model to explain the growth process of the CuO/ZnO heterojunction NRs. The reactions can be written as

(1) $$2{\mathrm{Cu}}^{2+}+3OH^{-}+NO_{3}^{-}\to Cu_{2}\left(NO_{3}\right){\left(OH\right)}_{3}$$

(2) $$2Cu_{2}\left(NO_{3}\right){\left(OH\right)}_{3}\to 4{\mathrm{CuO}+2\mathrm{NO}}_{2}+O_{2}+3H_{2}O.$$

The electrons in the valence band (VB) of ZnO transfer to the conduction band (CB) when the ZnO NRs were exposed under UV light. These electrons then transfer to the surface of ZnO nanorod and reacted with H_2_O or dissolved O_2_ in deionized water to generated OH^−^, which generates a large number of OH^−^ ions around ZnO NRs. Cu^2+^ ions gathered around ZnO NRs and formed Cu_2_(NO_3_)(OH)_3_ in the present of NO_3_^−^ (reaction (1)). In Dai’s [14] report, photochemical reaction deposited Ni(OH)_2_. Here, it was Cu_2_(NO_3_)(OH)_3_ instead of Cu(OH)_2_ formed on ZnO NRs in our experiment. Considering the low UV intensity, the formation of Cu_2_(NO_3_)(OH)_3_ can be attributed to the low OH^−^ concentration around ZnO NRs. Cu_2_(NO_3_)(OH)_3_ has lower solubility product (Ksp) than Cu(OH)_2_ and is preferred to produce in a low OH^−^ environment in the present of NO_3_^−^. In the photochemical reaction process, Cu_2_(NO_3_)(OH)_3_ nanoparticles is initially generated on upper part of ZnO NRs and facilitates subsequent nucleation and growth. Then these nanoparticles grow directionally and formed their own preferred sheet-like structures. Due to the spaces between ZnO NRs, Cu_2_(NO_3_)(OH)_3_ branches are obvious on the side surface of ZnO NRs, shown in the insets of Figure 1. Considering the low UV intensity of the lower part of the ZnO NRs and the gaps between the ZnO NRs, the growth of Cu_2_(NO_3_)(OH)_3_ branches are hindered at the lower part of the ZnO NRs. Moreover, photogenerated electrons have a fastest transport speed along the c axis. Thereby, Cu_2_(NO_3_)(OH)_3_ is mainly generated at the tips of ZnO NRs and forms bigger sheet-like structures. These results mean that the geometrical arrangement of ZnO NRs has a significant effect on the growth of Cu_2_(NO_3_)(OH)_3_ precursor. The sheet-like appearance of Cu_2_(NO_3_)(OH)_3_ is due to its natural layered crystal structure [38]. In the layered structure, octahedral copper forms layers of stoichiometry [Cu_2_(OH)_3_]^+^. NO_3_^−^ ions play a role of charge balance in the positive layers. NO_3_^−^ ions and hydroxyl groups are linked by hydrogen bonding in the octahedral copper layers. These Cu_2_(NO_3_)(OH)_3_ sheets would be converted to CuO by thermal decomposition (reaction (2)).

### 3.2. UV Detecting Performance of CuO/ZnO Heterojunction NRs

**Figure 7:**
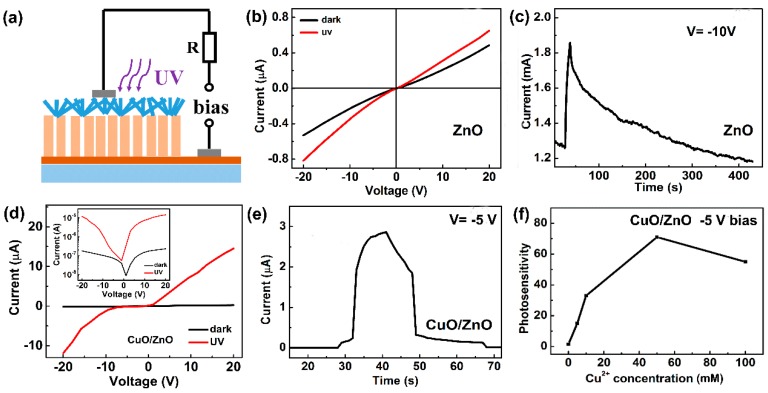
(**a**) The structure of CuO/ZnO heterojunction UV photodetectors. The voltages across the resistance is recorded to calculate the change of the photocurrents across the devices. The I-V characteristics (**b**) pure ZnO NRs and (**d**) the CuO/ZnO heterojunction in dark and under 365 nm light, the inset is the semi-log plot of the V-I curves. The single cycle in the I-t curves of (**c**) pure ZnO NRs and (**e**) the flower-like CuO/ZnO heterojunction NRs under UV illumination. (**f**) The photosensitivity of the CuO/ZnO heterojunction NRs devices prepared from Cu(NO_3_)_2_ solution with different Cu^2+^ ion concentrations. The UV irradiation time and the annealing temperature were fixed at 20 min and 300 °C, respectively.

The electrical properties and UV photo-response were measured using the sample synthesized in 50 mM Cu(NO_3_)_2_ aqueous solution and air annealed at 300 °C. Figure 7a displays the structure of the prepared CuO/ZnO heterojunction photodetector. The I-V characteristics of the ZnO and CuO/ZnO devices in dark and under UV light is displayed in Figure 7b,d. The pure ZnO NR device displays linear I-V curves, While the CuO/ZnO device displays a well-defined rectifying characteristic. The semi-log plot of the dark I-V curve is shown in the inset of Figure 7d. Figure S2 exhibits the linear I-V curves of Ag-CuO and AZO-ZnO, indicating good ohmic contacts. Hence, the rectifying behavior is not due to Schottky junctions at the electrodes but the CuO/ZnO p-n junction. Under 365 nm light illumination, both the two devices display UV response. Figure 7c,e show the time-resolved UV photocurrents of the pure ZnO and CuO/ZnO devices, respectively. The rise time (τ_r_) is the time for a 90% current rise and the decay time (τ_d_) is the time for a 90% current decay. The ZnO device displays a long rise time (9 s) and decay time (190 s). The CuO/ZnO device has a high response speed and the response times are much shorter (see Table 1), especially the decay time. It is interesting to see that the rising and falling edges of the CuO/ZnO device both consist of a fast process and a slow process, which will be discussed later. The response time of the fast process is less than 1 s, which is remarkably reduced as compared with that of the ZnO device. It is also seen that the fast process dominates the rising and falling edges and accounts for more than 70% of the photocurrent variations, which is large enough for fabricating fast response devices in real applications. For our CuO/ZnO device, the response speed is improved significantly in the reverse bias and the device shows a short response time, but in the forward current, a positive bias reduces the barrier height of CuO/ZnO p/n junction and the effect of oxygen adsorption is still obvious. The response speed is slower than that in the negative bias.

Photosensitivity, (I_ph_ − I_dark_)/I_dark_, is one of the important parameters for a photodetector, where I_ph_ and I_dark_ are photocurrent and dark current, respectively. All the performance parameters of the two devices are collected in Table 1. The photosensitivity of the flower-like CuO/ZnO NR device has been enhanced almost 50 times compared with the pure ZnO NRs device. According to these results, the photo-response performance of ZnO device has improved effectively by coating CuO on the surface of ZnO.

Also, the photosensitivity of the UV photodetectors is significantly affected by the CuO coverage degree on ZnO NRs which is related to the Cu^2+^ ion concentration during the photochemical deposition. The UV illumination time is fixed at 20 min and the CuO coverage degree is increasing with rising Cu^2+^ ion concentration. Figure 7f shows that the photosensitivity has a maximum value of 71 at –5 V bias when Cu^2+^ concentration is 50 mM, which is the optimal Cu^2+^ concentration in our experiment. As the Cu^2+^ concentration increases to 100 mM, the photosensitivity of the device deceases, because at a higher Cu^2+^ concentration, the CuO nanostructure layer has a larger thickness, which affects the light absorption of the CuO/ZnO device. Table 2 lists several parameters of our device and other CuO/ZnO UV photodetectors. Our device shows a high photosensitivity compared with other UV photodetectors based on CuO/ZnO nanostructures.

**Figure 8:**
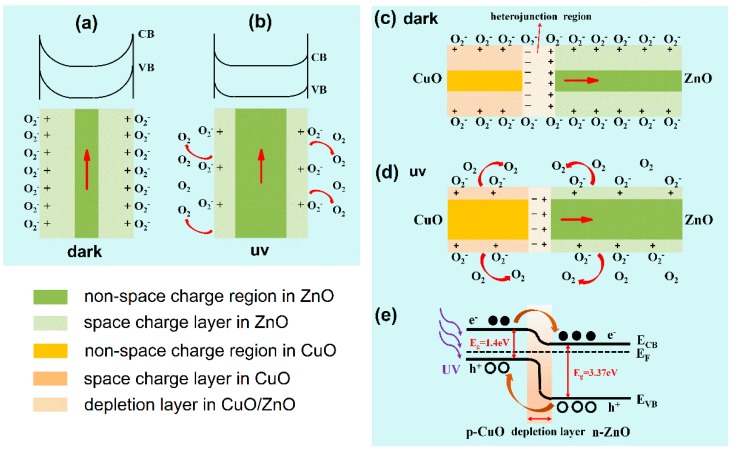
The barrier structure model of the ZnO NR UV photodetector under (**a**) dark and (**b**) UV environment. The barrier structure model of the ZnO/CuO heterojunction NR UV photodetector under (**c**) dark and (**d**) UV environment. (**e**) The energy diagram of the CuO/ZnO heterojunction NRs under UV illumination.

The principles of ZnO and CuO/ZnO photodetectors are described in Figure 8. For the ZnO device under the dark environment (Figure 8a), oxygen molecule adsorption traps the carriers in ZnO nanorods, forming O_2_^−^. Electrons are depleted from a space-charge layer near the surface and drive the nanorod into high resistivity state. When the device is illuminated by UV light (Figure 8b), there are a large number of photo-generated electron-holes pairs in the ZnO nanorods. Holes that are transferred to the surface of the ZnO nanorods react with O_2_^−^ and create O_2_ desorption. Thus, the carrier concentration of the device increases and the device switches to a low resistivity state. The adsorption and desorption process of oxygen molecules is slow, so the ZnO device has a long rise time and decay time (see Figure 7c). The adsorbed oxygen has been widely considered to explain the long photocurrent decay time in several nanostructured materials [39,40,41]. For the CuO/ZnO device, we can also observe slow rising and falling processes in the I-t curve (Figure 7e). The reason for this result is that the oxygen molecule adsorption and desorption process on the surface of the CuO/ZnO device is similar to that on the ZnO device. The difference is that there is a depletion layer at the interface between CuO and ZnO. Oxygen molecules cannot reach the interface and have little effect on the heterojunction. The very low dark current of the CuO/ZnO device is due to the heterojunction barrier (Figure 7c). Under the illumination of UV light, electrons in the CB of CuO transferred to ZnO and the holes in the VB of ZnO transferred to CuO (Figure 8e). Photogenerated electron-hole pairs are separated effectively under the heterojunction barrier, so the photosensitivity is high. The charge separation of a p-n junction is also very fast, so we can observe a very sharp rising edge in the I-t curve (Figure 7e). The explanation of the falling edge is similar to that of the rising edge.

## 4. Conclusions

In conclusion, we prepared CuO/ZnO heterojunction NRs using a facile photochemical deposition strategy. Cu(NO_3_)(OH)_3_/ZnO heterostructure NRs were synthesized as a precursor and then converted into CuO/ZnO heterostructure NRs after air annealing at a low temperature. The morphology of the CuO/ZnO heterostructure NRs is related to the growth conditions, including the Cu^2+^ ion concentration, UV illumination time, and annealing temperature. All the heterostructure devices showed well-defined rectified characteristic, and their UV detecting performances are improved compared with a pure ZnO NRs device. The CuO/ZnO NRs photodetectors fabricated with 50 mM Cu2+ ion concentration, 20 min UV illumination time, and 300 °C annealing temperature exhibit optimum device performance. The photosensitivity is increased by 50 times in contrast with the pure ZnO NR device. Especially, the response time of the fast process in the rising edge and falling edge is less than 1 s, which is remarkably reduced compared with that of the ZnO device. The facile and low-cost synthesis method not only can be used to fabricate CuO and NiO but also can be extended to the synthesis of the other metal oxide nanostructure on the ZnO NR surface.

## Figures and Tables

**Table 1:** The device performance of pure ZnO and CuO/ZnO UV photodetectors.

Samples	I_dark_ (A)	I_ph_ (A)	Photosensitivity	τ_r_ (s)	τ_d_(s)
ZnO	121	174	1.4	9	190
CuO/ZnO	0.002	0.142	71	6	7

**Table 2:** The device performance of our device and other CuO/ZnO UV photodetectors.

Samples	Photosensitivity	τ_r_ (s)	τ_d_ (s)
CuO/ZnO NWs [28]	2.4	>10	>10
CuO/ZnO NCs [29]	7.7	11.3	9
CuO/ZnO NWs [30]	61	—	—
CuO/ZnO [31]	2.2	4.2	5.2
This work	71	6	7

## Supplementary Materials

The following are available online at https://www.mdpi.com/2079-4991/9/5/790/s1, Figure S1: SEM images of as-grown ZnO NRs and ZnO NRs annealed at 400 °C in air, Figure S2: The I-V curves of Ag-CuO and AZO-ZnO.
