# Supplementary material for: CuO/ZnO Heterojunction Nanorod Arrays Prepared by Photochemical Method with Improved UV Detecting Performance
Source: Nanomaterials (Basel). 2019 May 23;9(5):790. doi: 10.3390/nano9050790 (PMC6566173; doi:10.3390/nano9050790)
Supplement: Supplementary file 1 [file nanomaterials-09-00790-s001.pdf]

Supporting Information for

CuO/ZnO Heterojunction Nanorod Arrays Prepared by Photochemical Method with Improved UV Detecting Performance

Jieni Li [1,2], Tingting Zhao [2], Mandar M. Shirolkar [2,3], Ming Li [2], Haiqian Wang [2,*] and Henan Li [4,*]

[1] International Collaborative Laboratory of 2D Materials for Optoelectronics Science and Technology of Ministry of Education, College of Physics and Optoelectronic Engineering, Shenzhen University, Shenzhen 518060, China; jnli91@szu.edu.cn

[2] Hefei National Laboratory for Physical Sciences at the Microscale, University of Science and Technology of China, Hefei, Anhui 230026, China; zhaott27@mail.ustc.edu.cn (T.Z.); mmshirolkar@gmail.com (M.M.S.); seagullc@ustc.edu.cn (M.L.)

[3] Symbiosis Center for nanoscience center and nanotechnology, Symbiosis International, Deemed University, Lavale, Pune 412115, India

[4] College of Electronic Science and Technology, Shenzhen University, Shenzhen 518060, China

* Correspondence: hqwang@ustc.edu.cn (H.W.); henan.li@szu.edu.cn (H.L.)

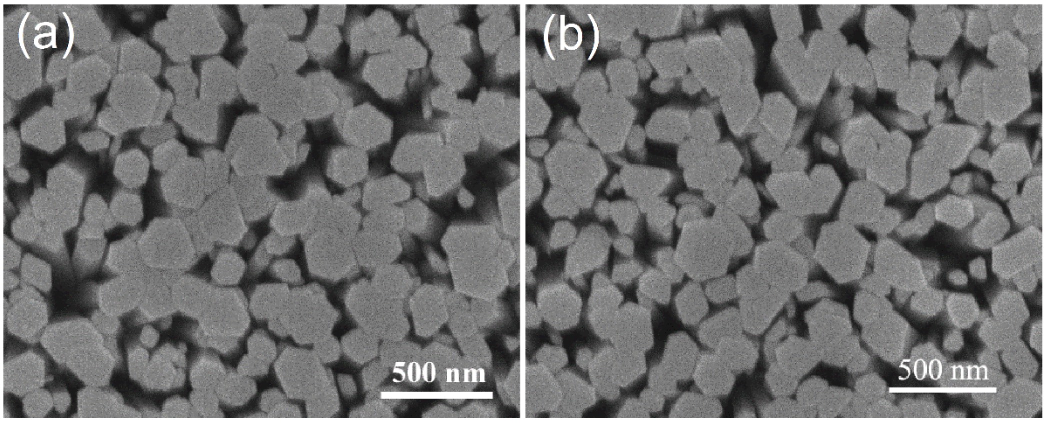

Figure S1. the surface image of (a) the as-grown ZnO nanorod arrays (NRs) and (b) the 400 °C annealing ZnO NRs. Annealing at 400 °C has no effect on the surface morphology of ZnO NRs.

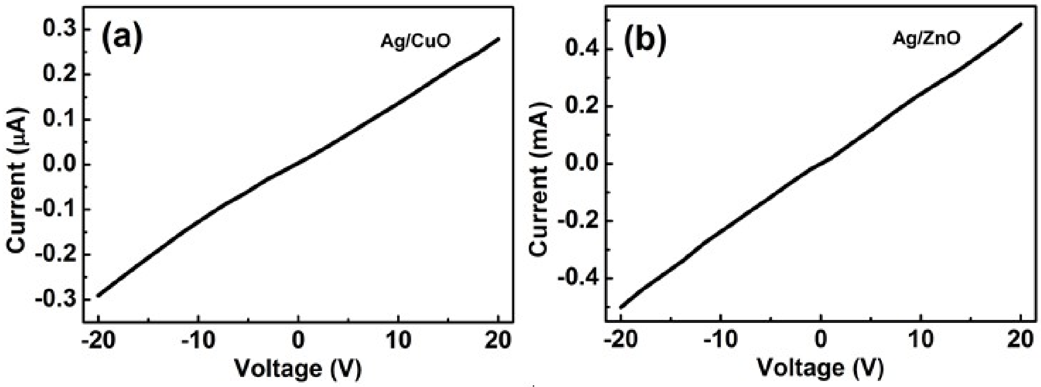

Figure S2. The V-I curves of (a) Ag-CuO and (b) AZO-ZnO. Both V-I curves shows a good ohmic contact.
